# The combined effect of socioeconomic status and metabolic syndrome on depression: the Korean National Health and Nutrition Examination Survey (KNHANES)

**DOI:** 10.1186/s12889-020-08778-3

**Published:** 2020-05-04

**Authors:** B. Kim, E. Y. Park

**Affiliations:** Division of Cancer Prevention & Early Detection, National Cancer Control Institute, National Cancer Center, 323, Ilsan-ro, Ilsandong-gu, Goyang-si, Gyeonggi-do 10408 Republic of Korea

**Keywords:** Social environment, Metabolism, Depression, Mental health

## Abstract

**Background:**

Depression shows different patterns depending on socioeconomic status (SES) and metabolic syndrome (MS). However, the nature of this association remains poorly understood. The aim of this study was to examine whether the combination of MS and lower SES was associated with the prevalence of depression, based on data from the Korea National Health and Nutrition Examination Survey (KNHANES).

**Methods:**

Data were obtained from a cross-sectional study of 24,102 adults (> 19 years of age) who participated in the KNHANES during 2008–2013 and for whom MS and depression data were available. MS was defined using the diagnostic criteria of the modified National Cholesterol Education Program Adult Treatment Panel III. Measure of depression was ascertained from self-reports of physician diagnosis. Multiple logistic regression analysis was used to evaluate the association between depression and MS as well as SES (alone and in combination).

**Results:**

Overall, 622 of the 24,102 subjects (2.6%) met the criteria for depression. The prevalence of depression was associated with MS, a lower high-density lipoprotein cholesterol level, an elevated triglyceride level, a lower education level, and a lower household income. Participants with MS and a low SES had a higher likelihood of depression than those without MS and a high SES (odds ratio [OR] = 4.180 for low education level and OR = 3.994 for low household income level).

**Conclusions:**

This study suggests that the combination of SES and MS may play an important role in depression, which has implications for healthcare policy and depression management.

## Background

Depression is a common mood disorder, the prevalence of which increased during the twentieth century [1]. Depression is especially associated with increased cardiovascular morbidity and all-cause mortality [2], and it may be linked to an elevated risk of poor health outcomes through its association with low socioeconomic status (SES) and metabolic syndrome (MS) [3]. However, little is understood about mechanisms that may account for adverse health outcomes associated with depression.

Previous reports have speculated that depression may be linked to adverse health outcomes through an association with the metabolic syndrome [1, 4, 5]. MS is a complex disorder characterized by a cluster of metabolic, anthropometric, and hemodynamic abnormalities. The National Cholesterol Education Program (NCEP) Adult Treatment Panel III (ATP III) [6] defined MS as the presence of at least three of the following: abdominal obesity, elevated triglyceride (TG) levels, decreased high-density lipoprotein cholesterol (HDL-C) levels, hypertension, and hyperglycemia. Previous studies have reported associations between depression and several MS components, including larger waist circumference [1, 3, 7]; higher levels of glucose [7, 8], blood lipids [9, 10], and TG [1, 3]; higher blood pressure (BP) [1, 11, 12]; and lower HDL-C levels [1, 3]. Accumulating evidence suggests that depression is not simply a comorbidity of MS. Instead, it has been suggested that the association between obesity and mood disorders represents a distinct condition known as metabolic–mood syndrome [13–16]. However, many questions remain unanswered regarding the above associations.

Lower socioeconomic status (SES) is associated with higher odds of having depression [17, 18]. Beset by growing national and international inequalities in socioeconomic status (SES) has come into focus as a significant determinant of depression [17]. The effect of SES on depression is an important theme, and this supports previous studies which illustrate the negative association between SES and depression [19–21]. In South Korea, the “Establish the National Health Plan 2020” project, published in 2011 [22], aimed to achieve health equity and promote health across all stages of life and reduce the gap in health status across different SES levels [23]. This is highly relevant because the incidences of both depression in Korea differ according to SES [24, 25]. In a study of community dwellers on Jeju Island, South Korea [26], the prevalence of depressive symptoms differed according to monthly income and education status. Kahng and Kwon reported that individuals with a relatively low SES, such as those with a low income, low education level, and female sex, exhibited high levels of depression (Kang and Kwon, 2008).

Most previous studies focused on the relationship between MS and depression. However, the results of these studies were conflicting [1, 7, 18], possibly because of differences in SES. Moreover, no published studies have explored the combined effects of SES and MS on depression. Therefore, the aim of this study was to examine whether the combination of SES and MS was associated with the prevalence of depression in Korean men and women who participated in the Korean National Health and Nutrition Examination Survey (KNHANES).

## Methods

### Study population

KNHANES is a cross-sectional survey of a nationally representative sample of the civilian, noninstitutionalized population in South Korea. This population-based survey has a multistage sampling design and includes three assessments: health interview, health examination, and nutrition survey. KNHANES has contributed to the development and evaluation of health policies and programs, facilitated the establishment of reference values (such as growth charts and dietary references) for the Korean population, and supported health research [27].

Data used in this study were derived from the 2008 to 2013 KNHANES data, which were stratified according to age, sex, and geographic area. We used data from the health interview and health examination. In total, 53,829 individuals participated in KNHANES from 2008 to 2013: 9744 in 2008, 10,533 in 2009, 8958 in 2010, 8518 in 2011, 8058 in 2012, and 8018 in 2013. Study participants were at least 20 years of age. Of these, 26,393 subjects with missing data for depression (*n* = 472) or MS (*n* = 1819) were excluded. The final study population therefore consisted of 24,102 adults.

All procedures contributing to this study complied with the ethical standards of the relevant national and institutional committees on human experimentation and with the Helsinki Declaration of 1975, as revised in 2008. All procedures involving human subjects were approved by the Korea Centers for Disease Control and Prevention Institutional Review Board, and all participants signed a written informed consent form.

### Data collection

As previously described in detail [27], KNHANES is conducted by four specialized research teams, each of which consists of eight experts, including nurses, nutritionists, and students majoring in public health. The selected professional investigator was placed at the investigation site after completing 1 month of training and conducted interviews with participants using a structured questionnaire. The following information was collected: presence of physician-diagnosed hypertension, diabetes mellitus, stroke, heart disease (including myocardial infarction or angina), osteoporosis, osteoarthritis, and depression; current or previous back pain; medication for hypertension, diabetes mellitus, or hyperlipidemia; and sociodemographic and lifestyle data, including age, sex, marital status, education, household income, smoking status, alcohol consumption, and physical activity. Participants who smoked < 100 cigarettes in their life were classified as never smokers; the remainder were categorized as current or former smokers. Individuals consuming ≥12 alcohol-containing drinks per year were considered alcohol drinkers. Physical activity and history of chronic diseases were evaluated by yes or no responses to relevant questions. Participants were divided into monthly household income quartiles: low (1,200,000 won), medium (1,210,000–4,300,000 won), and high (> 4,310,000 won). Participants were also classified by educational level: less than elementary school, middle school, high school, or college or more). The results of standardized health examinations conducted at local community health centers and clinics were used to obtain anthropometric data (height, weight, and waist circumference). Body mass index (BMI) was calculated as weight in kilograms divided by height in meters squared. Waist circumference was measured at the midpoint between the 12th rib and anterior iliac spine. BP was measured on the right arm using an automatic sphygmomanometer with participants in the sitting position, after resting for 5 min. Average systolic blood pressure (SBP) and diastolic blood pressure (DBP) values of at least two repeated measurements were calculated. Blood samples were collected after overnight fasting to measure fasting blood sugar (FBS), TG, total cholesterol, HDL-C, and high-sensitivity C-reactive protein levels.

### Metabolic syndrome definition

According to the revised NCEP ATP III [6] definition, a person may be diagnosed with MS when they meet three or more of these criteria: 1) abdominal obesity, determined by a large waist circumference (> 90 cm for men; > 80 cm for women), according to the International Obesity Task Force criteria for the Asia–Pacific population [26–28]; 2) TG level ≥ 150 mg/dL or use of medication to reduce TG levels; 3) low HDL-C (< 40 mg/dL for males; < 50 mg/dL for females) or use of medication for hypercholesterolemia; 4) hypertension (SBP ≥ 130 mmHg or DBP > 85 mmHg) or use of antihypertensive medication; or 5) FBS ≥ 100 mg/dL or use of medication for hyperglycemia.

### Assessment of depression

As described in detail previously [29], depression was measured using these two screening questions, for which “yes” or “no” answers were solicited: 1) “In your lifetime, have you ever had depression?” and 2) “Have you ever been diagnosed with depression by a physician?” If the response to the second question was yes, the age at first depression diagnosis was solicited. Based on these questions, we defined two outcome measures for depression: (1) self-reported depression and (2) self-reported physician-diagnosed depression. For statistical analysis, depression was defined as the presence of either of these two outcomes.

### Statistical analysis

KNHANES data were combined for all years from 2008 to 2013. Descriptive data are presented as weighted means or percentages with standard errors. Multiple logistic regression analysis for a complex sampling design was performed using PROC SURVEYLOGISTIC in SAS to evaluate the association between depression and SES, as well as MS (alone and in combination). Model 1 was adjusted for age (year, continuous) and sex. Model 2 was adjusted for sex, age, education level (less than elementary school, middle school, high school, or college or more), household income (low, middle, or high), marital status (married, unmarried, or divorced/widowed/separated), physical activity (yes or no), smoking status, alcohol consumption (non-drinker, ≤ 2–4 times/month, 2–3 times/week, or ≥ 4 times/week), and chronic disease status (yes or no). Model 3 added history of depression as a covariate and included the confounding variables of model 2. Combined effects represent the combination of SES level and MS, with SES defined by education level or household income. The following eight groups were defined according to education level and the presence or absence of MS: 1) college or more without MS, 2) college or more with MS, 3) high school without MS, 4) high school with MS, 5) middle school without MS, 6) middle school without MS, 7) less than elementary school without MS, and 8) less than elementary school with MS. Similarly, the following five groups were defined according to household income and the presence or absence of MS: 1) high income without MS, 2) high income with MS, 3) middle income without MS, 4) middle income with MS, 5) low income without MS, and 6) low income with MS. We also examined whether the combination of SES and MS was associated with the prevalence of depression in men and women. Data analyses were performed using SAS version 9.3 software with a survey procedure (SAS Institute, Inc., Cary, NC). Statistical significance was defined as a value of *p* < 0.05.

## Results

Table 1 shows the descriptive characteristics of the study population, as well as the risk (odds ratios [OR] and 95% confidence intervals [95% CI]) of depression according to these characteristics. Depression was identified in 622 of the 24,102 subjects, representing a 2.6% prevalence of depression. Compared to the subgroup of participants with depression, the overall population were more likely to be older and married, have a higher education level and household income, and not have a physician-diagnosed history of depression. In contrast, participants with depression were more likely to be female and non-drinkers of alcohol; be divorced/widowed/separated; and have at least one chronic disease. The prevalence of MS was 20.1% in the overall population and 3.2% in participants with depression. The overall population was more likely to have an elevated TG level and lower FBS, whereas participants with depression were more likely to have a lower HDL-C and larger waist circumference.

**Table 1:** Prevalence and odds ratio for being depression by general characteristics of study population with metabolic syndrome

Characteristics	Prevalence of depression			
	Total (weighted %)	Yes (weighted %)	OR(95% CI)	*p*-value
	*n* = 24,102(100.0)	*n* = 622(2.6)		
Gender				
Male	10,228(51.0)	128(1.2)	1.00	<.0001
Female	13,874(49.0)	494(3.2)	2.80(2.30–3.51)	
Age group (years)				
19–29	4369(24.3)	79(1.5)	1.00	<.0001
30–39	6616(26.0)	132(1.7)	1.11(0.80–1.54)	
40–49	6643(27.3)	166(2.4)	1.57(1.14–2.15)	
50–59	6474(22.4)	245(3.3)	2.19(1.63–2.96)	
Education level				
College or more	9269(38.1)	114(1.0)	1.00	<.0001
High school	10,259(45.1)	249(2.1)	2.12(1.63–2.77)	
Middle schools	2312(8.9)	104(4.0)	4.14(2.99–5.74)	
Elementary school or less	2236(7.8)	154(6.3)	6.62(4.94–8.88)	
House income				
High	8260(33.2)	132(1.3)	1.00	<.0001
Middle	13,531(57.9)	340(2.1)	1.71(1.33–2.20)	
Low	2041(9.0)	142(5.8)	4.87(3.58–6.62)	
Marital status				
Married	17,775(68.0)	407(2.0)	1.00	<.0001
Unmarried	4860(26.6)	94(1.6)	0.82(0.63–1.08)	
Divorced/Widowed/separated	1419(5.4)	119(7.4)	3.93(3.06–5.05)	
Body mass index				
18.5–22.9	10,096(41.3)	229(1.9)	1.00	0.03
< 18.5	1163(5.0)	32(1.9)	1.01(0.65–1.57)	
≥ 23.0	12,793(53.7)	360(2.4)	1.26(1.03–1.53)	
Smoking status				
non-smokers	14,261(53.1)	393(2.3)	1.00	0.02
< 5pack	727(3.4)	27(3.5)	1.49(0.96–2.34)	
≥ 5pack	9074(43.5)	199(1.9)	0.79(0.65–0.94)	
Alcohol consumption				
Non-drinkers	4830(17.6)	191(3.7)	1.00	0.003
≤ 2-4times/month	13,772(57.5)	328(2.0)	0.53(0.43–0.66)	
2-3times/week	3990(18.2)	66(1.5)	0.39(0.28–0.54)	
≥ 4times/week	1424(6.6)	36(2.0)	0.55(0.37–0.81)	
Physical activity				
No	18,558(76.9)	476(2.2)	1.00	0.39
Yes	5509(23.1)	144(2.0)	0.91(0.73–1.13)	
History of depression diagnosed by a doctor				
No	23,312(96.9)	283(0.9)	1.00	<.0001
Yes	790(3.1)	339(41.4)	1.71(1.38–2.10)	
Prevalence of chronic diseases				
No	22,026(92.8)	464(1.8)	1.00	<.0001
Yes	2076(7.2)	158(6.7)	3.81(3.02–4.82)	
Metabolic syndrome				
No	19,175(79.9)	447(1.9)	1.00	<.0001
Yes	4927(20.1)	175(3.2)	1.70(1.38–2.11)	
Waist circumference (cm)				
< 90 (< 80 for women)	16,956(72.2)	348(1.8)	1.00	<.0001
≥ 90 (≥80 for women)	7146(27.8)	274(3.2)	1.83(1.51–2.22)	
Triglyceride level (mg/dL)				
< 150	17,771(72.8)	437(2.0)	1.00	0.01
≥ 150	6331(	185(2.7)	1.34(1.078–1.66)	
Blood pressure^*^				
Normal	18,804(77.6)	498(2.2)	1.00	0.28
Abnormal	5298(22.4)	124(2.0)	0.88(0.69–1.11)	
HDL cholesterol level (mg/dL)				
≥ 40 (≥50 for women)	14,539(62.9)	297(1.7)	1.00	<.0001
< 40 (< 50 for women)	9563(37.1)	325(3.1)	1.85(1.53–2.24)	
FBS (mg/dL)				
< 100	18,811(78.4)	460(2.0)	1.00	0.01
≥ 100	5291(21.6)	162(2.8)	1.37(1.10–1.72)	

Normal: systolic < 130 mmHg and diastolic < 85 mmHg; abnormal: systolic ≥130 mmHg and diastolic 85 mmHg

ORs for depression decreased with increasing SES (Table 1). ORs for depression according to education level were 6.62 (95% CI 4.94–8.88) for elementary school or less, 4.144 (95% CI 2.99–5.74) for middle school, and 2.12 (95% CI 1.63–2.77) for high school, compared with college or more as the reference (OR 1.0). ORs for depression according to household income level were 4.87 (95% CI 3.58–6.62) for low income and 1.71 (95% CI 1.33–2.20) for middle income, compared with high income as the reference. Participants with MS had a higher prevalence of depression than those without MS. MS (OR = 1.70), larger waist circumference (OR = 1.83), higher HDL-C (OR = 1.85), and higher FBS (OR = 1.37) were all associated with depression. BP was not associated with depression.

Table 2 presents the results of logistic regression analyses of the association between depression and MS, each MS component, education level, and household income, after controlling for sex, age, education level, income, marital status, physical activity, smoking status, alcohol consumption, and chronic disease (model 2). MS was associated with an increased likelihood of depression (OR = 1.34, 95% CI 1.05–1.72). Additionally, two MS components were significantly associated with an increased likelihood of depression: lower HDL-C (OR = 1.40, 95% CI 1.12–1.74) and higher TG (OR = 1.35, 95% CI 1.06–1.71). There was also a non-statistically significant trend toward an association between depression and a larger waist circumference (OR = 1.21, 95% CI 0.92–1.60) and higher FBS (OR = 1.23, 95% CI 0.95–1.58), but no association was detected between BP and depression. Similar results were observed in model 3 (adjusted for the confounding factors of model 2 and a history of depression). Household income and education level were inversely associated with depression in all models (*p* < 0.0001).

**Table 2:** Odds ratios for being depression by metabolic syndrome and each of its components and SES (primarily education level and household income)

Characteristics	Model1^a^		Model2^b^		Model3^c^	
	OR(95% CI)	*p*-value	OR(95% CI)	*p*-value	OR(95% CI)	*p*-value
Waist circumference (cm)						
< 90 (< 80 for women)	1.00	0.0002	1.00	0.18	1.00	0.10
≥ 90 (≥80 for women)	1.49 (1.21–1.82)		1.21 (0.92–1.60)		1.30 (0.95–1.78)	
Triglyceride level (mg/dL)						
< 150	1.00	<.0001	1.00	0.02	1.00	0.09
≥ 150	1.61 (1.27–.2.03)		1.35 (1.06–1.71)		1.26 (0.97–1.65)	
Blood pressure^a^						
Normal	1.00	0.42	1.00	0.13	1.00	0.32
Abnormal	0.90 (0.70–1.16)		0.82 (0.63–1.06)		0.87 (0.65–1.15)	
HDL cholesterol level (mg/dL)						
≥ 40 (≥50 for women)	1.00	<.0001	1.00	0.003	1.00	0.0004
< 40 (< 50 for women)	1.52 (1.25–.1.86)		1.40 (1.12–1.74)		1.55 (1.22–1.99)	
FBS (mg/dL)						
< 100	1.00	0.01	1.00	0.11	1.00	0.09
≥ 100	1.36 (1.07–1.73)		1.23 (0.95–1.58)		1.26 (0.96–1.65)	
Metabolic syndrome						
No	1.00	<.0001	1.00	0.02	1.00	0.003
Yes	1.65 (1.30–2.09)		1.34 (1.05–1.72)		1.51 (1.15–1.98)	
Education level						
College or more	1.00	<.0001	1.00	<.0001	1.00	<.0001
High school	2.10 (1.60–2.79)		1.77 (1.34–2.33)		1.52 (1.13–2.06)	
Middle schools	4.02 (2.76–5.87)		2.86 (1.93–4.24)		2.73 (1.79–4.15)	
Elementary school or less	6.20 (4.26–9.02)		3.72 (2.49–5.54)		3.16 (2.06–4.85)	
House income						
High	1.00	<.0001	1.00	<.0001	1.00	<.0001
Middle	1.73 (1.35–2.23)		1.33 (1.02–1.72)		1.45 (1.09–1.94)	
Low	4.66 (3.42–6.36)		2.54 (1.84–3.51)		2.95 (2.05–4.24)	

^a^Model1: adjusted for age

^b^Model2: adjusted for age, education level, income, marital status, moderate physical activity more than, smoking status, alcohol consumption, prevalence of chronic diseases

^c^Model3: adjusted for age, education level, income, marital status, moderate physical activity more than, smoking status, alcohol consumption, prevalence of chronic diseases, history of depression

To compare the association between depression and MS, MS components, and SES in males and females, logistic regression analyses with adjustment for confounding variables were performed (Table 3). Household income and education level were inversely associated with depression in both sexes. Among females, MS was associated with depression in all models. Two MS components were associated with an increased likelihood of depression in females but not in males: larger waist circumference and lower HDL-C. We also found that in females, the interaction term for education level and MS was significant, whereas the interaction term for household income level and MS was not significant; neither interaction term was significant in males (see Additional file 1).

**Table 3:** Odds ratios for being depression by metabolic syndrome and each of its components and SES (primarily education level and household income) in male and female

Characteristics	Male						Female					
	Model1^a^		Model2^b^		Model3^c^		Model1^a^		Model2^b^		Model3^c^	
	OR (95% CI)	*p*-value	OR (95% CI)	*p*-value	OR (95% CI)	*p*-value	OR (95% CI)	*p*-value	OR (95% CI)	*p*-value	OR (95% CI)	*p*-value
Waist circumference (cm)												
< 90 (< 80 for women)	1.00	0.75	1.00	0.92	1.00	0.43	1.00	<.0001	1.00	0.03	1.00	0.004
≥ 90 (≥80 for women)	1.08 (0.67–1.48)		0.97 (0.57–1.67)		0.78 (0.42–1.44)		1.70 (1.36–2.14)		1.46 (1.05–2.04)		1.78 (1.20–2.63)	
Triglyceride level (mg/dL)												
< 150	1.00	0.02	1.00	0.01	1.00	0.15	1.00	0.0004	1.00	0.07	1.00	0.16
≥ 150	1.66 (1.09–2.53)		1.77 (1.13–2.76)		1.45 (0.88–2.39)		1.63 (1.24–2.13)		1.30 (0.98–1.73)		1.25 (0.91–1.70)	
Blood pressure^a^												
Normal	1.00	0.23	1.00	0.24	1.00	0.32	1.00	0.93	1.00	0.45	1.00	0.76
Abnormal	0.76 (0.48–1.20)		0.76 (0.48–1.21)		0.77 (0.46–1.29)		1.02 (0.75–1.38)		0.89 (0.65–1.22)		0.95 (0.68–1.32)	
HDL cholesterol level (mg/dL)												
≥ 40 (≥50 for women)	1.00	0.05	1.00	0.22	1.00	0.09	1.00	0.0002	1.00	0.002	1.00	0.001
< 40 (< 50 for women)	1.53 (1.00–2.35)		1.34 (0.84–2.16)		1.59 (0.94–2.68)		1.52 (1.22–1.90)		1.47 (1.16–1.86)		1.57 (1.20–2.04)	
FBS (mg/dL)												
< 100	1.00	0.27	1.00	0.33	1.00	0.13	1.00	0.02	1.00	0.27	1.00	0.43
≥ 100	1.29 (0.82–2.04)		1.29 (0.78–2.12)		1.50 (0.89–2.53)		1.39 (1.06–1.83)		1.18 (0.88–1.57)		1.14 (0.83–1.57)	
Metabolic syndrome												
No	1.00	0.08	1.00	0.11	1.00	0.10	1.00	<.0001	1.00	0.03	1.00	0.01
Yes	1.51 (0.95–2.38)		1.49 (0.92–2.42)		1.58 (0.92–2.71)		1.76 (1.35–2.29)		1.40 (1.04–1.87)		1.55 (1.13–2.13)	
Education level												
College or more	1.00	0.01	1.00	<.0001	1.00	<.0001	1.00	<.0001	1.00	0.01	1.00	0.06
High school	2.50 (1.32–4.92)		2.18 (1.14–4.16)		2.17 (1.08–4.36)		1.91 (1.42–2.57)		1.55 (1.14–2.09)		1.36 (0.98–1.89)	
Middle schools	6.94 (3.26–14.77)		4.75 (2.29–9.84)		7.36 (3.33–16.30)		3.17 (2.02–4.98)		2.08 (1.30–3.34)		1.90 (1.15–3.14)	
Elementary school or less	16.80 (8.12–34.79)		10.94 (5.24–22.85)		10.78 (4.73–24.61)		4.07 (2.63–6.30)		2.26 (1.41–3.60)		1.93 (1.15–3.24)	
House income												
High	1.00	<.0001	1.00	<.0001	1.000	<.0001	1.00	<.0001	1.00	<.0001	1.00	<.0001
Middle	1.45 (0.85–2.46)		0.98 (0.56–1.71)		0.914 (0.49–1.71)		1.83 (1.39–2.41)		1.47 (1.10–1.95)		1.71 (1.24–2.35)	
Low	7.07 (4.01–12.46)		2.82 (1.57–5.06)		2.793 (1.46–5.34)		3.75 (2.66–5.28)		2.25 (1.56–3.26)		2.651 (1.72–4.10)	

^a^Model1: adjusted for age

^b^Model2: adjusted for age, education level, income, marital status, moderate physical activity more than, smoking status, alcohol consumption, prevalence of chronic diseases

^c^Model3: adjusted for age, education level, income, marital status, moderate physical activity more than, smoking status, alcohol consumption, prevalence of chronic diseases, history of depression

**Fig. 1:**
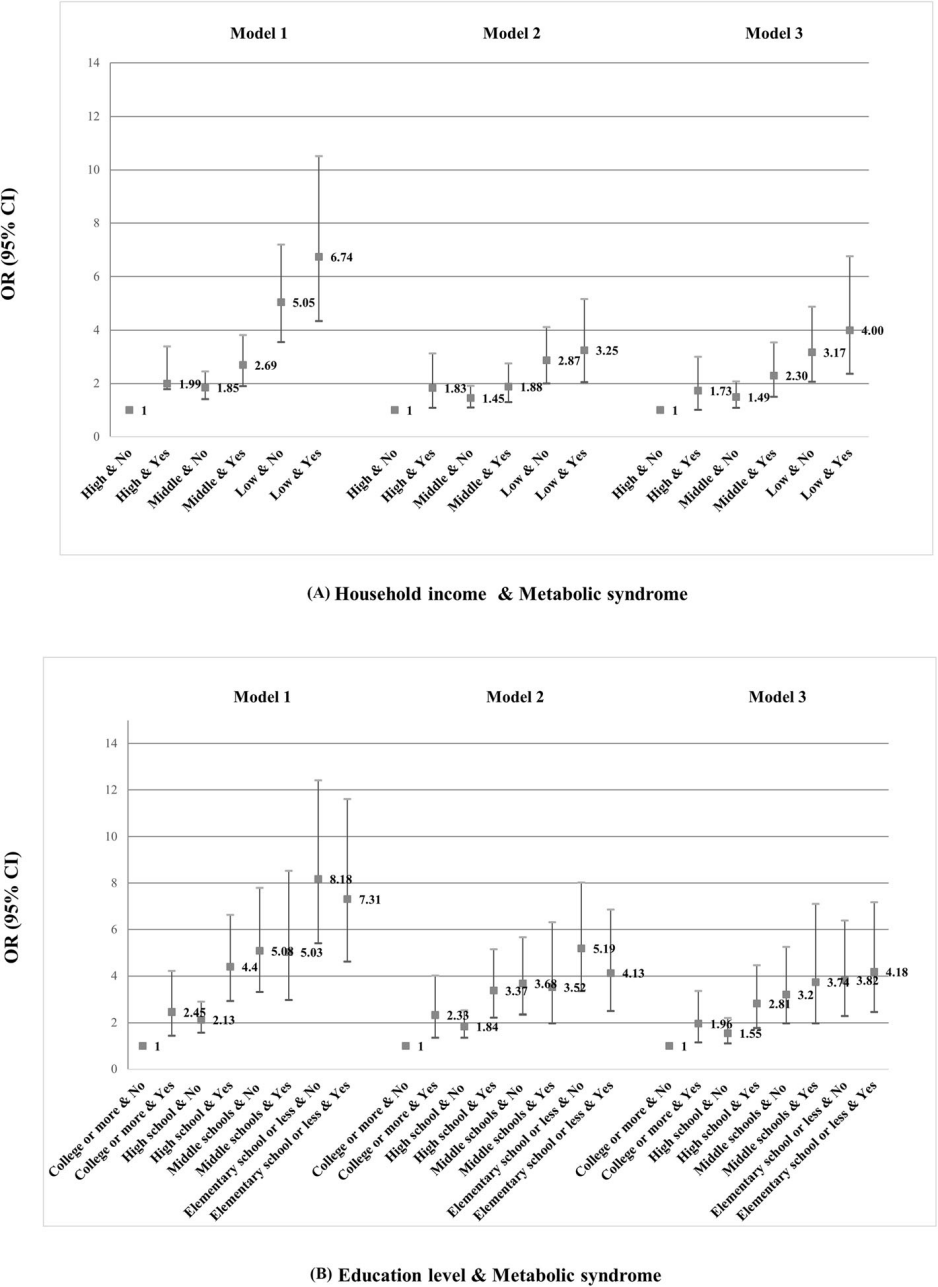
Combined effects of metabolic syndrome and SES for depression. **a** Household income and Metabolic syndrome **b** Primarily Education level and Metabolic syndrome. Model1: adjusted for gender, age. Model2: adjusted for gender, age, education level, income, marital status, moderate physical activity more than, smoking status, alcohol consumption, prevalence of chronic diseases. Model3: adjusted for gender, age, education level, income, marital status, moderate physical activity more than, smoking status, alcohol consumption, prevalence of chronic diseases, history of depression

ORs for depression for the combination of SES (household income or education level) and MS are shown in Fig. 1. After adjusting for covariates, the OR for depression was 4.18 (95% CI 2.36–6.75) for the combination of low education level (elementary school or less) and MS, compared with high education level (college or more) and no MS as the reference (model 3). Similarly, the OR for depression was 4.00 (95% CI 2.36–6.75) for the combination of low household income and MS, compared with high household income and no MS as the reference (model 3). In all models, the combination of lower SES (household income or education level) and MS was associated with the presence of depression. Furthermore, the combination of education level and MS was more strongly associated with depression in males than in females, as represented by higher ORs (Table 4).

**Table 4:** Combined effects of metabolic syndrome and SES (primarily education level and household income) for depression in male and female

Characteristics	Male						Female					
	Model1^a^		Model2^b^		Model3^c^		Model1^a^		Model2^b^		Model3^c^	
	OR (95% CI)	*p*-value	OR (95% CI)	*p*-value	OR (95% CI)	*p*-value	OR (95% CI)	*p*-value	OR (95% CI)	*p*-value	OR (95% CI)	*p*-value
Education level & Metabolic syndrome												
College or more & No	1.00	<.0001	1.00	<.0001	1.00	<.0001	1.00	<.0001	1.00	0.0001	1.00	0.02
College or more & Yes	2.26 (0.76–6.74)		2.18 (0.73–6.48)		1.96 (0.66–5.83)		3.40 (1.80–6.41)		3.16 (1.63–6.13)		2.55 (1.28–5.10)	
High school & No	2.41 (1.13–5.15)		2.12 (1.01–4.49)		2.13 (0.91–4.99)		2.02 (1.45–2.81)		1.69 (1.20–2.37)		1.45 (0.10–2.10)	
High school & Yes	6.30 (2.61–15.21)		4.90 (2.07–11.57)		4.29 (1.75–10.53)		4.00 (2.51–6.38)		2.96 (1.78–4.91)		2.53 (1.43–4.48)	
Middle schools & No	9.23 (3.63–23.46)		6.31 (2.62–15.23)		8.43 (3.25–21.84)		4.14 (2.56–6.71)		2.80 (1.70–4.61)		2.39 (1.35–4.23)	
Middle schools & Yes	10.11 (3.52–29.04)		6.98 (2.30–21.12)		11.84 (3.62–38.68)		3.82 (2.00–7.30)		2.46 (1.20–5.08)		2.45 (1.21–4.96)	
Elementary school or less & No	25.31 (10.79–59.40)		15.60 (6.59–36.90)		13.87 (5.22–36.84)		5.25 (3.25–8.49)		3.16 (1.88–5.30)		2.31 (1.25–4.28)	
Elementary school or less & Yes	19.81 (7.44–52.74)		13.97 (5.10–38.25)		14.74 (4.69–46.28)		5.28 (3.15–8.84)		2.74 (1.54–4.83)		2.81 (1.52–5.22)	
Household income & Metabolic syndrome												
High^a^ No	1.00	<.0001	1.00	0.001	1.00	0.001	1.00	<.0001	1.00	<.0001	1.00	<.0001
High^a^ Yes	1.10 (0.41–2.96)		1.27 (0.47–3.44)		0.91 (0.34–2.45)		2.69 (1.55–4.67)		2.36 (1.34–4.15)		2.30 (1.22–4.34)	
Middle^a^ No	1.24 (0.67–2.29)		0.87 (0.46–1.63)		0.67 (0.31–1.43)		2.09 (1.54–2.83)		1.71 (1.25–2.33)		1.91 (1.33–2.74)	
Middle^a^ Yes	2.14 (1.07–4.29)		1.54 (0.72–3.29)		1.60 (0.66–3.88)		3.02 (2.02–4.50)		2.16 (1.94–3.36)		2.69 (1.64–4.41)	
Low^a^ No	6.70 (3.49–12.84)		2.81 (1.45–5.45)		2.62 (1.22–5.63)		4.22 (2.85–6.23)		2.67 (1.76–4.05)		2.94 (1.77–4.87)	
Low^a^ Yes	8.85 (3.96–19.78)		3.48 (1.43–8.43)		3.01 (1.13–8.01)		5.93 (3.54–9.91)		3.13 (1.82–5.39)		4.05 (2.18–7.53)	

^a^Model1: adjusted for age

^b^Model2: adjusted for age, education level, income, marital status, moderate physical activity more than, smoking status, alcohol consumption, prevalence of chronic diseases

^c^Model3: adjusted for age, education level, income, marital status, moderate physical activity more than, smoking status, alcohol consumption, prevalence of chronic diseases, history of depression

## Discussion

We found that SES and MS, both alone and in combination, were associated with the prevalence of depression. Lower SES increased the prevalence of depression, and this relationship was strongest among individuals with the lowest level of education or household income. MS and two MS components (lower HDL-C and higher TG) were significantly associated with an increased likelihood of depression, after adjusting for other potential risk factors. There was also a trend toward a positive association between depression and two other MS components (increased waist circumference and FBS). Additionally, participants with the combination of MS and lower SES had an increased the prevalence of depression, when compared with individuals with a higher SES and no MS.

Previous studies reported that depression is more prevalent among people with a lower SES [18, 30–32]. Our results are in agreement with these observations. Data from the Alameda County Study, a community-based longitudinal study of psychological and social factors and their role in health and well-being in almost 7000 adults from Alameda County, California, demonstrated a graded relationship between SES and both prevalent and incident depression [33]. Among participants who were not depressed at the beginning of the study, those with low education (0–8 years) were increase risk when compared with those who have 12 years or more of education (OR = 1.86, 95% CI 1.36–2.55) [34]. In another the Alameda County Study, which defined SES by household income tertiles, yielded results similar to ours: 19% of lower-income respondents reported numerous depressive symptoms, but only approximately 11% of higher-income respondents reported these symptoms [33]. A meta-analysis evaluating socioeconomic inequality in depression noted that low-SES individuals had an elevated the prevalence of depression (OR = 1.81, 95% CI 1.57–2.10) [18]. These findings suggest lower SES may be associated with risks of depression and should be considered a major risk factor for depression.

Our results also showed an association between depression and MS. These findings are consistent with those of previous studies [5, 35–37]. A longitudinal study found that participants with MS at baseline were 2-fold more likely to develop depressive symptoms during follow-up than healthy matched controls [5]. In a cohort of healthy middle-aged females in the Healthy Women Study, MS predicted the development of depressive symptoms over a 6-year follow-up period [35]. In another population-based study, females with MS during childhood had higher levels of depressive symptoms in adulthood, and the severity of these symptoms increased in proportion to the duration of MS [36]. In 5698 individuals from the Northern Finland 1966 Birth Cohort Study, MS was not associated with depression when assessed at the age of 31 years [38]. We also found that depression was associated with several MS components (waist circumference, HDL-C, TG, and FBS), although only the associations with lower HDL-C and higher TG remained significant after controlling for covariates in logistic regression analysis. Several previous studies evaluated associations between MS components and depression [3, 35, 37]. The Netherlands Study of Depression and Anxiety found that higher Inventory of Depressive Symptomatology scores were associated with a higher number of MS-related abnormalities, larger waist circumference, higher HDL-C, higher TG, and lower SBP [3]. Of five MS components evaluated in a study conducted in the United Kingdom, central obesity, high TG, and low HDL-C were predictive of depressive symptoms [35]. In a study from India, waist circumference was positively correlated with the severity of depressive symptoms measured using the Hamilton Depression Rating scale (*r* = 0.291, *p* = 0.003) [37]. Whether MS components are more predictive of depression than MS itself is unclear [39]. Further research is required to confirm our findings and define the role of MS components as risk factors for depression.

In the present study, we found that the risk of depression was 4-fold higher among participants with MS and a lower SES than in those without MS and a higher SES. We observed similar trends in all three models. Two previous studies reported the prevalence of depression and its comorbidities according to SES [40, 41]. In the MultiCare Cohort Study, lower SES was associated with a higher prevalence of chronic conditions, such as cardiovascular and metabolic disorders (CMDs), as well as the composite outcome of depression, anxiety, somatoform disorders, and pain. The number of CMDs was associated with the level of education (− 0.17 CMDs for medium level and − 0.26 CMDs for high level, when compared with a low education level) and income (− 0.27 CMDs per unit on a logarithmic scale). SES of patients with CMDs was inversely related to the prevalence of depression in both males and females, although the association was stronger in females [40]. In the other study, which was a population-based study of adult females in the United States Buffalo–Niagara region, the prevalence of depression plus obesity was higher in more educated women than in less educated women (OR = 2.15, 95% CI 1.27–3.62) [41]. By contrast, the combination of education level and MS was more strongly associated with depression in males than in females in our study. These findings suggest the existence of important sex differences for the combined role of education level and MS on the development of depression. It is also possible that men underestimate their negative affective states because of differences in cognitive appraisal, accessibility of memories, or reliance on implicit beliefs, stereotypes, or cultural differences [42]. This, in turn, may mask the true nature of the associations between SES, MS and depression. Sex differences in the association between SES, MS and depression may also have physiological mechanisms, but this requires further investigation [7]. Numerous studies have reported the prevalence of depression among participants with a lower SES or MS [18, 30–34]. However, little is known about the combined effects of SES and MS on depression. To our knowledge, there have been no previous reports regarding the association between depression and the combination of SES and MS.

The current study suggests that SES and MS are an important factor in depression, however, the mechanism underlying its role remains unclear. The relation between SES and MS on depression may be at least partly explained by autonomic nervous system changes. In animal models, neuroendocrine system responses to SES have included hyperexcitation of the sympathetic system, hypersecretion of cortisol, and increased visceral fat. These changes are similar to those found in MS, and they have been suggested to occur in depression as well [43]. Furthermore, subordinate rats (which are a model for social stress, as may occur in humans with a low SES) have elevated levels of plasma insulin and leptin and display overeating behavior [25, 44]. Depression has also been associated with changes in inflammatory and hemostatic markers, including increased platelet aggregation, fibrinogen levels, and white blood cell counts [1, 45, 46]. As inflammatory and hemostatic processes also play important roles in MS, depression may be linked to MS and its components through these processes [1]. Further research in this area is warranted.

The present study had some limitations. First, we were unable to determine whether there is a causal relationship between depression and MS and/or low SES, because of the study’s cross-sectional design. Prospective studies are required to establish whether a causal relationship exists. Second, information bias was unavoidable because the study was based on a questionnaire. Third, the presence of depression was based on self-reported data. However, the lifetime prevalence of self-reported depression (2.6%) in this population was similar to the 2.7% prevalence reported in a previous Korean study in which depression was assessed using a well-validated questionnaire [47]. Our findings should be verified using more comprehensive assessments of depression. Fourth, our focus on five metabolic factors may represent an oversimplification of the extremely complex pathophysiologic processes leading to atherosclerosis. High cholesterol and BP may interact synergistically with other, non-conventional risk factors in the development of atherosclerosis.

Despite these limitations, this study has several strengths, including the use of data from a large population of apparently healthy subjects with a low prevalence of chronic disease (92.8% of the overall study population had no chronic disease). Additionally, because of considerable variation in MS components among individuals, we were able to analyze various MS components, in addition to MS itself. Finally, this study included an assessment of several variables, such as disease history, health behavior, education, and household income, as well as the interaction between SES and MS.

## Conclusions

In conclusion, this study showed that SES and MS, both alone and in combination, were associated with the prevalence of depression. Our findings indicate the importance of the combined effect of SES and MS in depression. It remains a task of future research to determine whether the combination of SES and MS was associated with the prevalence of depression across various populations. An in-depth understanding of the combination of SES and MS that are present in individuals with depression can provide basic data that would help to public health strategies for reduce the prevalence of depression.

## Supplementary information

**Additional file 1:Table S1.** Odds ratio (95% CI) and interactive effects of metabolic syndrome and SES (primarily education level and household income) on depression.

## Data Availability

All data files are available from the Korea Centers for Disease Control and Prevention database through the following URL: https://knhanes.cdc.go.kr/knhanes/sub03/sub03_02_02.do. Any person, including an international researcher who signs up for membership, can obtain raw data from this website. However, the data access process and user manual are only written in Korean.

## Abbreviations

KNHANES: the Korean National Health and Nutrition Examination Survey
SES: Socioeconomic Status
MS: Metabolic Syndrome
NCEP: National Cholesterol Education Program
ATP III: Adult Treatment Panel III
TG: Triglyceride
HDL-C: High-density lipoprotein Cholesterol
BP: Blood Pressure
BMI: Body mass index
SBP: Systolic Blood Pressure
DBP: Diastolic Blood Pressure
FBS: Fasting Blood Sugar
OR: Odds ratios
95% CI: 95% confidence intervals
CMD: Metabolic disorders
