# Supplementary material for: The combined effect of socioeconomic status and metabolic syndrome on depression: the Korean National Health and Nutrition Examination Survey (KNHANES)
Source: BMC Public Health. 2020 May 4;20:617. doi: 10.1186/s12889-020-08778-3 (PMC7197185; doi:10.1186/s12889-020-08778-3)
Supplement: Supplementary file 1 — Additional file 1:Table S1. Odds ratio (95% CI) and interactive effects of metabolic syndrome and SES (primarily education level and household income) on depression. [file 12889_2020_8778_MOESM1_ESM.pdf]

Table S1. Odds ratio(95% CI) and interactive effects of metabolic syndrome and SES (primarily education level and household income) on depression.

Characteristics	OR(95% CI)			
	Male		Female	
	Crude	Adjusted	Crude	Adjusted
Model 1: Education level interaction				
Metabolic syndrome (MS)				
No	1.000	1.000	1.000	1
Yes	1.88 (0.64-5.50)	1.26 (0.32-4.96)	1.90 (1.02-3.53)	1.28 (0.61-2.64)
Education level				
College or more	1.000	1.000	1.000	1.000
High school	3.09 (1.62-5.91)	2.67 (1.38-5.15)	1.90 (1.42-2.55)	1.33 (0.95-1.87)
Middle schools	9.45 (4.17-21.44)	10.20 (4.38-23.76)	3.26 (2.23-4.78)	1.93 (1.13-2.52)
Elementary school or less	26.09 (11.84-57.50)	16.68 (6.49-42.89)	4.79 (3.28-7.01)	2.04 (1.12-3.71)
Interaction term of education level*MS (*p*-value)	0.05	0.31	0.0003	0.05
Model 2: Household income level interaction				
Metabolic syndrome (MS)				
No	1	1	1	1
Yes	1.78 (0.93-3.44)	1.52 (0.68-3.39)	1.34 (0.85-2.12)	1.21 (0.72-2.01)
Household income level				
High	1.000	1.000	1.000	1
Middle	1.34 (0.76-2.34)	0.88 (0.44-1.74)	1.90 (1.44-2.50)	1.81 (1.31-2.51)
Low	7.17 (3.62-14.22)	3.03 (1.30-7.06)	4.28 (2.94-6.22)	3.32 (2.02-5.44)
Interaction term household income level*MS (*p*-value)	0.87	0.89	0.13	0.24

Adjusted for age, education level, household income level, marital status, moderate physical activity more than, smoking status, alcohol consumption, prevalence of chronic diseases, history of depression
